# Investigating the effect of AS03 adjuvant on the plasma cell repertoire following pH1N1 influenza vaccination

**DOI:** 10.1038/srep37229

**Published:** 2016-11-16

**Authors:** J. D. Galson, J. Trück, D. F. Kelly, R. van der Most

**Affiliations:** 1 Oxford Vaccine Group, Department of Paediatrics, University of Oxford and the NIHR Oxford Biomedical Research Center, Oxford, United Kingdom; 2 Paediatric Immunology, University Children’s Hospital Zürich, Switzerland; 3 GSK Vaccines, Rixensart, Belgium

## Abstract

Influenza pandemics require rapid deployment of effective vaccines for control. Adjuvants such as AS03 improve vaccine immunogenicity, but this mechanism is poorly understood. We used high-throughput B cell receptor sequencing of plasma cells produced following AS03-adjuvanted and non-adjuvanted 2009 pandemic H1N1 vaccination, as well as pre-pandemic seasonal influenza vaccination to elucidate the effect of the adjuvant on the humoral immune response. By analyzing mutation levels, it was possible to distinguish sequences from cells that were recently activated from naïve B cells from those that were activated by memory recall. We show that the adjuvant functions through two mechanisms. First, the adjuvant stimulates increased activation of naïve B cells, thus reducing immune interference with previous vaccine responses. Second, the adjuvant is able to increase the adaptability of the recalled cells to give improved specificity to the new vaccine antigen. We thus show how AS03 enhances pH1N1 immune responses, and reduces immune interference.

Influenza virus causes seasonal outbreaks of clinical influenza, and has been responsible for four pandemics over the last 100 years^1^. While seasonal outbreaks are associated with mutation of the haemagglutinin (HA) protein on the viral surface to escape neutralization by antibodies generated in previous exposures, pandemics result from the introduction of completely new viruses into populations, where there is little pre-existing immunity to that virus^2^. The latest influenza pandemic arose in 2009, and was caused by a swine-origin H1N1 virus (pH1N1), and resulted in an estimated 300,000 deaths within the first 12 months^3^. The pre-pandemic 2008/2009 seasonal trivalent influenza vaccines (TIV) did contain an H1N1 strain (A/Brisbane/59/2007), but this differed considerably at the structural level from the pandemic strain, with 24 AA differences at key antigenic sites^4^, and thus offered only limited heterotypic protection^5^,^6^.

The capacity to rapidly develop and manufacture effective vaccines in large quantities is key in combating influenza pandemics. Adjuvants can enhance vaccine immunogenicity, allowing a reduction in the quantity of antigen per dose and a consequent increase in the number of doses that can be manufactured in a given time-period. Many pH1N1 vaccines were therefore formulated with an oil-in-water adjuvant (AS03 or MF59), and these conferred greater immunogenicity than non-adjuvanted vaccines, even when using just a quarter of the antigen dose^7^,^8^. Despite the success of these adjuvants, the details of their mode of action in the context of influenza vaccine are still poorly understood.

AS03 and MF59 enhance innate immune responses by increasing antigen uptake and presentation in the local tissue. This in turn leads to increased CD4 T cell, and B cell responses^9^,^10^. For pandemic influenza vaccination, this suggests that the adjuvant could improve B cell responses by either increasing activation of naïve B cells, or by increasing the activation and adaptation of pre-existing memory B cells generated through infection or immunization with seasonal influenza from earlier years to become specific towards the pandemic strain^11^. In a previous study, we investigated the effect of AS03 on the pH1N1 vaccine response, and also the effect of TIV priming on the subsequent pH1N1 response^8^. This study indicated that prior TIV administration decreased both the humoral and T cell response to pH1N1 vaccine, but adjuvanting the pH1N1 vaccine helped to overcome this effect^8^. Such a finding is potentially consistent with the adjuvant working by either stimulating more naïve B cell activation, or by increasing adaptation of pre-existing memory B cells, but gives no mechanistic insight.

Understanding the mode of action of the adjuvant can be helped by studying the properties of the plasma cells produced in response to the vaccine. Khurana *et al*. used phage display libraries, and surface plasmon resonance to determine binding locations, and affinity of the antibodies produced in response to both adjuvanted and non-adjuvanted pandemic influenza vaccines^12^,^13^. They found that the antibodies produced in response to the adjuvanted vaccine displayed a greater diversity of binding targets, had a shift away from targeting the conserved stem region of HA towards the more variable head region, and had a greater avidity than those produced in response to the non-adjuvanted vaccine^12^,^13^. These results suggested that the adjuvant mainly functioned by stimulating more of a naïve vaccine response by activating B cells targeting different epitopes, and not through more extensive diversification of pre-existing memory cells.

An increased understanding of the repertoire of plasma cells produced in response to vaccination could potentially be gained by sequencing their B cell receptor (BCR) heavy chain variable regions^14^,^15^. Knowing the exact nucleotide sequences allows determination of mutation numbers, which can be used to distinguish between the plasma cells activated from naïve B cells vs. pre-existing memory B cells. Other features of the repertoire, such as diversity, and isotype subclass usage can also be used to provide further insight into the immune mechanisms that generated the plasma cells. If sequential samples are taken following repeat vaccinations from a single participant, it is also possible to directly identify and characterize memory recall by looking for shared sequences between the two samples^16^.

Here, we utilized the same samples from our previous study, which investigated the effect of TIV priming, and AS03 adjuvant on the pH1N1 response for high-throughput plasma cell BCR sequencing^8^. Plasma cell samples were sequenced 7 days following administration of the pH1N1 vaccine either with or without AS03 adjuvant (Fig. 1). Day 7 was chosen as this represents the peak of the antigen-specific plasma cell response to influenza vaccination, with previous studies demonstrating 33–80% of plasma cells isolated at this time to be specific to the vaccine^17^,^18^. In the participants where TIV was given four months prior to the pH1N1 vaccination, the plasma cell repertoire was also sequenced 7 days following administration of this vaccine. Obtaining paired samples following both TIV and pH1N1 vaccination allowed direct investigation of memory recall between these two vaccines. Our findings support the notion that the adjuvant functions through a combination of both increasing naïve B cell activation, and also by increasing the adaptation of pre-existing memory B cells for greater specificity towards the pandemic strain.

## Results

### Adjuvant improves the humoral and T cell response to pH1N1 vaccination

**Figure 1:**
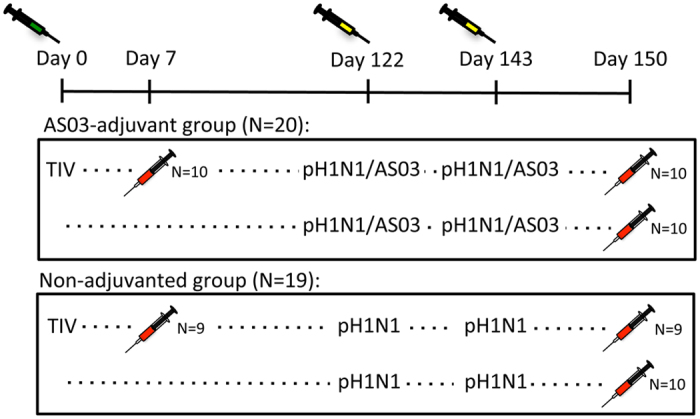
Study design. Participants were randomized to receive either AS03-adjuvanted, or non-adjuvanted pH1N1 vaccine. Two doses of pH1N1 vaccine were given 3 weeks apart, and blood taken for plasma cell repertoire analysis 7 days following the second pH1N1 vaccine. In both the AS03 adjuvanted and non-adjuvanted groups, half of the participants were administered TIV four months prior to their pH1N1 vaccination. Blood was also taken for plasma cell repertoire analysis 7 days following the TIV vaccination.

**Figure 2:**
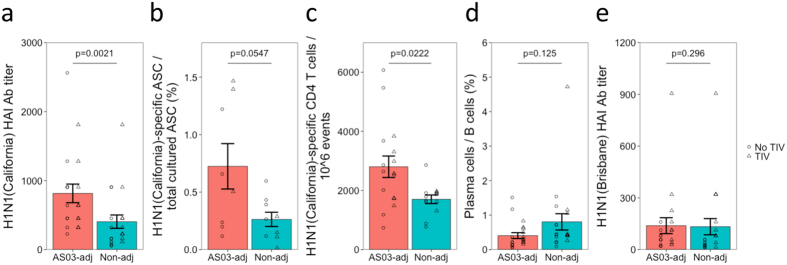
Serological and cellular measures of pH1N1 vaccine response taken 7 days following vaccination. (**a**) H1N1(California)-specific HAI antibody titer, (**b**) H1N1(California)-specific memory B cells determined by ELISpot, (**c**) H1N1(California)-specific activated CD4 T cells determined by intracellular cytokine staining and flow cytometry, (**d**) plasma cells determined by flow cytometry, and (**e**) H1N1(Brisbane)-specific HAI antibody titer. Measurements were carried out for all 39 participants, except for ELISpot, which was only conducted for a subset of 17 participants. Participants were grouped according to whether they received an AS03-adjuvanted or non-adjuvanted vaccine. Individual data points are shown, with the shape representative of whether the participant received the seasonal TIV prior to pH1N1 vaccination. Shown are the mean values ± SEM. P values show the result from a two-sided t-test.

Of the 118 participants in the original study^8^, 39 were used for plasma cell repertoire sequencing in this study (Fig. 1). Here we focused solely on the response 7 days following the pH1N1 vaccine - more comprehensive analyses can be found in the original study^8^. The humoral and T cell responses in the subset of 39 participants reflected those from the whole cohort. All participants were seropositive for 2009 H1N1(California) by 7 days following their first dose of pH1N1 vaccine. The haemagglutination inhibition (HAI) titer, the number of 2009 H1N1(California)-specific memory B cells, and the number of activated 2009 H1N1(California)-specific CD4 T cells following pH1N1 vaccination was greater in the group receiving the adjuvanted vaccine compared to non-adjuvanted vaccine (Fig. 2a–c)^8^. Plasma cell frequency, and heterotypic seroconversion to the 2007 H1N1(Brisbane) viral strain in the TIV vaccine were similar between the adjuvanted and non-adjuvanted vaccine groups (Fig. 2d,e)^8^.

Nineteen (10 in the adjuvanted group, and 9 in the non-adjuvanted group) of the participants had received the 2009 seasonal TIV vaccine four months previously. While the data in the original study indicated that prior TIV vaccination reduced the subsequent response to pandemic vaccine, this potentially confounding variable was not considered for the analyses presented here^8^. The effect of TIV was small in relation to the effect of the adjuvant, and the reduced group sizes here make it unlikely that any differences would be detectable. Furthermore, the number of participants receiving TIV vs. not receiving TIV was similar in the non-adjuvanted and adjuvanted groups.

### Sequencing the plasma cell repertoire following pH1N1 and TIV vaccination

To further investigate the B cell responses, plasma cells were isolated at day 7 following both pH1N1 and TIV (where applicable) vaccination for BCR sequencing (Fig. 1 and Fig. S1). On average, 3,433 (range: 464–10,651) plasma cells were isolated from each sample (Table S1). Plasma cell numbers were similar in the adjuvant and non-adjuvanted groups, and greater after TIV vaccination (mean numbers of 3,003; 2,754; and 4,577 respectively). A total of 19,101,557 raw sequencing reads were obtained, which reduced to 9,505,320 following sequence quality control and filtering steps. Sequences from each sample were clustered using a previously described method to group together sequences that are derived from the same clonal lineage, and also those that may have arisen due to PCR and sequencing error^15^,^19^. On average, 2,353 (range: 627–4,528) clusters were generated for each sample. The number of clusters was closely related to the number of input cells, giving a 1:1.2 ratio of clusters to cells (Fig. S2a).

We have previously described some simple quality control measures that can be applied to BCR sequence data to detect outliers^20^, and these measures were applied to the current dataset (Fig. S2b,c). Principal component analysis of global repertoire diversity, V gene mutation and CDR3 length, can be used to detect biological outliers, while principal component analysis of V gene usage profiles can be used to detect outliers due to technical artifacts. No outliers were found in the current dataset using either of these measures (Fig. S2b,c).

### Plasma cells isolated following vaccination are enriched for vaccine specificity

**Figure 3:**
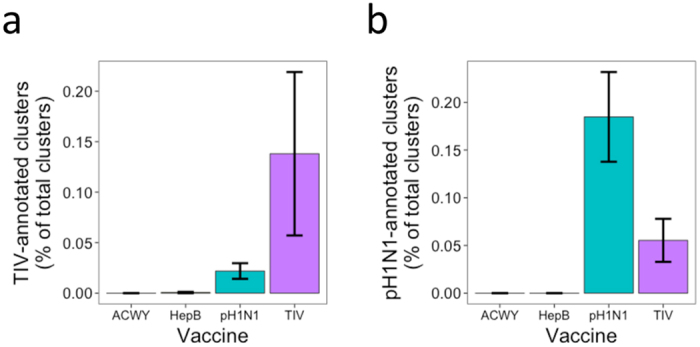
Specificity of plasma cell sequence clusters following vaccination with different antigens. (**a**) Percent of total clusters annotated as having specificity to 2007–2009 seasonal influenza based on comparison to previous data. (**b**) Percent of total clusters annotated as having specificity to pH1N1 based on comparison to previous data. Bars show mean values ± SEM. N = 9 for Meningococcal ACWY vaccination, 14 for Hepatitis B vaccination, 39 for pH1N1 vaccination, and 19 for TIV vaccination.

While not all plasma cells produced 7 days following vaccination will be specific to the vaccinating antigen, previous studies have shown vaccine-specific plasma cells to be highly enriched at this time^17^,^18^. To verify that the plasma cells isolated in this study were enriched for vaccine-specificity, a database of previously described pH1N1-specific, and 2007–2009 seasonal influenza-specific BCR sequences were collated from the literature, and compared to our dataset. In total, 182 seasonal influenza-specific^21^, and 126 pH1N1-specific sequences^22^,^23^,^24^,^25^ were obtained from the literature. These sequences were used to annotate 74 clusters in our dataset as having seasonal influenza specificity, and 206 clusters as having pH1N1 specificity (based on sharing the same CDR3 AA region as sequences within the cluster). Clusters annotated as specific to seasonal influenza were most common after TIV vaccination, although some were still found after pH1N1 vaccination (Fig. 3a,b). The converse was true for clusters annotated as specific to pH1N1. The pH1N1 and seasonal influenza-specific sequences were also compared to BCR sequence datasets of plasma cells collected following both Hepatitis B 20,26 and Meningococcal ACWY^15^ vaccination, but no matches were found (Fig. 3a,b).

### Using mutation number to distinguish clusters derived from naïve B cell activation vs. memory recall

**Figure 4:**
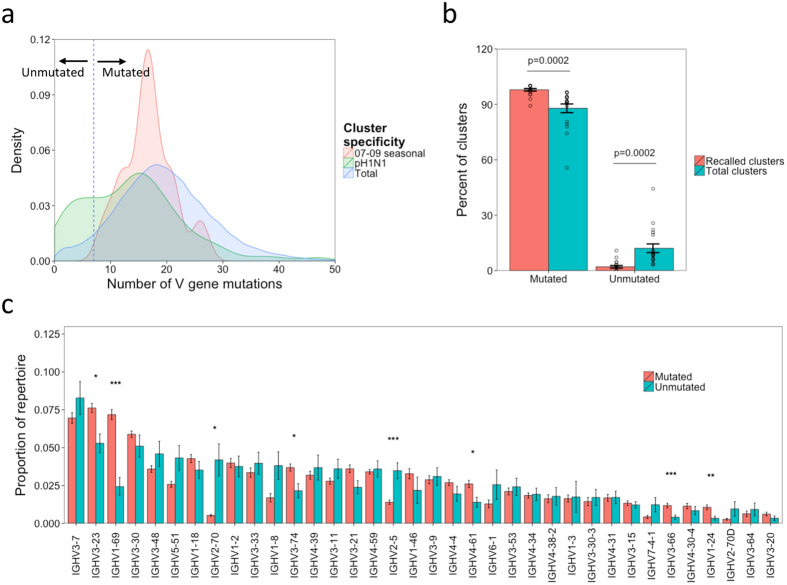
Distinguish clusters derived from naïve B cell activation from clusters derived from memory recall following pH1N1 vaccination. (**a**) Density histogram showing the number of mutations of either total clusters following pH1N1 vaccination, or clusters annotated as having specificity to either pH1N1, or seasonal influenza based on comparison to previous data. Dotted vertical line separates clusters with less than 7 mean mutations (unmutated) from those with at least 8 mean mutations (mutated). (**b**) Clusters present following both TIV and pH1N1 vaccination were identified (recalled clusters), and the percent of these classed as either mutated or unmutated (based on the 7 mutation cutoff) was determined following pH1N1 vaccination. Percent of total clusters that were mutated or unmutated was then determined for comparison. P value shows the result from a paired two-sided t-test. (**c**) For each sample, the proportion of both mutated and unmutated clusters utilizing different V gene segments was determined. Bars show mean values ± SEM. Shown are the V genes with a proportion above 0.005 in at least one of the groups. Comparisons were performed using a two-sided paired t-test. *p < 0.01, **p < 0.001, ***p < 0.0001.

The finding that some seasonal influenza-specific clusters are found following pH1N1 vaccination suggests that some B cells activated following TIV vaccination are subsequently recalled following pH1N1 vaccination. The number of V gene mutations of the seasonal influenza-specific and pH1N1-specific clusters was analyzed following pH1N1 vaccination, to try and distinguish between clusters closely related to germline (and are thus more likely to have been recently activated from naïve B cells), compared to clusters which have diverged more from germline (and are thus more likely to have arising from memory recall). All of the seasonal influenza-specific clusters were highly mutated (minimum of 9 V gene mutations), whereas the pH1N1-specific clusters had a bimodal distribution with some highly mutated, and some with very little mutation (Fig. 4a). As the seasonal-influenza specific clusters must derive from memory recall, but the pH1N1 clusters can derive either from memory recall or naïve activation, this information was used to place a mutation cutoff of 7 mutations (corresponding to approximately 2.5% of mutated bases) to distinguish unmutated clusters more likely to have arisen from naïve B cells compared to mutated clusters more likely to have arisen from memory recalled B cells (although we cannot discount that some unmutated clusters may also be derived from memory recall). While 2.5% mutation is more than is expected in naïve IgM B cells^15^, we are here interested in class-switched plasma cells recently derived from these naïve B cells, so more mutation would be expected^27^. A threshold of 2.5% mutation also ensures that sequencing errors are not overinterpereted as somatic hypermutation in the mutated clusters.

As an independent measure to validate the 2.5% mutation threshold, specific recall between the TIV to pH1N1 vaccines was determined by combined analysis of these two datasets. Co-clustering the data from the TIV and pH1N1 datasets for each participant revealed that on average 52 clusters were shared between their TIV and pH1N1 plasma cell datasets, equating to 1% of the total number of clusters present in the two datasets. As the probability of the same CDR3 being produced during two independent recombination events during the lifetime of a single individual is practically zero, it is likely that these shared clusters represent memory recall of the same B cell lineage^16^,^28^. On average, 98% of these recalled clusters were classed as mutated following pH1N1 vaccination, compared to 88% of total clusters (p  =  0.0002; Fig. 4b), which is what we would expect if mutated clusters represent those derived from memory recall.

### Unmutated and mutated clusters have distinct V gene usage profiles

To further investigate the properties of the mutated and unmutated clusters following pH1N1 vaccination, for each sample, the proportion of the repertoire of these clusters comprised by different V genes was determined. V gene usage differed dramatically between the mutated and unmutated clusters, regardless of whether the dataset was split based on receipt of adjuvanted or non-adjuvanted vaccine, or previous receipt of TIV (Fig. 4c and Fig. S3). Usage of IGHV1-69, IGHV3-23, IGHV3-74, IGHV4-61, IGHV3-66 and IGHV1-24 was greater in the mutated clusters, while usage of IGHV2-70 and IGHV2-5 was greater in the unmutated clusters. No V gene usage differences between mutated and unmutated clusters were observed when carrying out the same analysis on a control dataset of plasma cells previously collected in the absence of any specific immune stimulus (Fig. S4)^20^,^26^.

While V gene usage alone cannot be used to determine what epitope a sequence will bind to, it is well documented that some V genes are preferentially used in the creation of BCR’s with certain specificities, indicating that the mutated and unmutated clusters may target distinct HA epitopes^14^. In the context of influenza vaccine, IGHV1-69 is associated with the production of HA stem-binding (rather than head-binding) cross-reactive antibodies^29^,^30^. Usage of IGHV1-69 showed the most significant difference between the mutated and unmutated clusters; IGHV1-69 was three times more abundant in the mutated compared to unmutated clusters.

### Adjuvant increases the proportion of unmutated clusters specific to the vaccine

**Figure 5:**
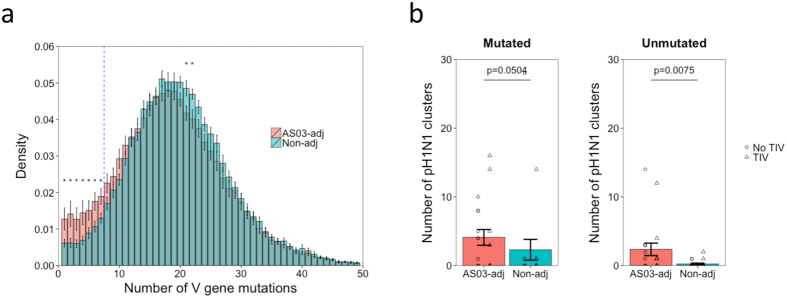
Effect of adjuvant on stimulation of naïve vs. recalled cells. (**a**) Distribution of total clusters with different mean numbers of V gene mutations, split by vaccine group. Dotted vertical line separates clusters defined as unmutated or mutated (threshold of 7). *p < 0.05; two-sided t-test. Bars show mean values ± SEM. (**b**) The number of mutated or unmutated clusters annotated as having specificity for pH1N1 based on comparison to previous data was determined. Bars show mean values ± SEM. Individual data points are shown, with the shape representative of whether the participant received the seasonal TIV prior to pH1N1 vaccination. P values show the result from a two-sided Mann-Whitney U test.

Following on from the observation that the unmutated and mutated clusters appear to represent distinct populations which may represent plasma cells more recently activated from naïve B cells versus those derived from memory recall, the effect of the adjuvant on the ratio of unmutated to mutated clusters was determined. The mean number of mutations was slightly lower in the participants receiving the adjuvanted compared to non-adjuvanted pH1N1 vaccine (18.5 vs. 19.8 respectively), and this was caused by a relative increase in the number of unmutated compared to mutated clusters in the adjuvant group (Fig. 5a). On average, in the adjuvant group, 10.6% of clusters were unmutated, compared to 5.8% in the non-adjuvanted group. Following TIV vaccine, where it is expected that the response is more dominated by memory recall than the response to pH1N1 vaccine, only 3.5% of clusters were unmutated (Fig. S5).

While the unmutated clusters represent a minority of the total number of clusters, they are more enriched for pH1N1-specificity (based on comparison to previously described sequences) than the mutated clusters. On average, 0.42% of unmutated clusters were annotated as pH1N1-specific, compared to 0.15% of the mutated clusters. Comparing the clusters annotated as pH1N1-specific between the vaccine groups showed that more pH1N1-specific clusters were present in the adjuvant compared to the non-adjuvanted group (Fig. 5b). While this difference remained regardless of whether unmutated or mutated clusters were considered, it was more significant for the unmutated (p  =  0.0075; 9-fold increase) compared to the mutated (p  =  0.0504; 2-fold increase) clusters. These data suggest that the increase in unmutated B cells stimulated by the adjuvanted vaccine also relates to an increase in vaccine-specific B cells.

### Adjuvant increases adaptation of recalled cells

**Figure 6:**
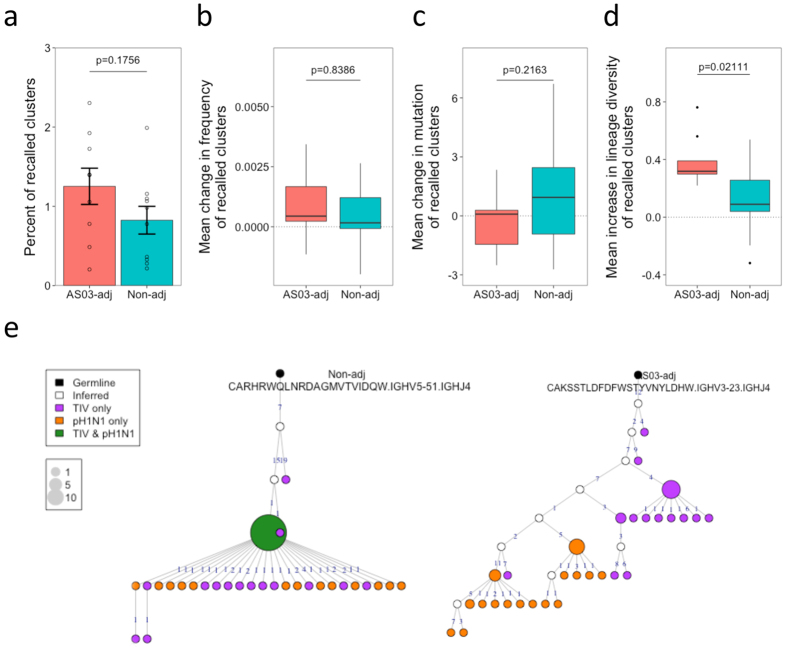
Measuring memory recall from TIV to pH1N1 vaccination. (**a**) For the 19 participants who were given a TIV vaccine four months prior to receiving the pH1N1 vaccine, the number of clusters that could be found in the plasma cell sequence data following both vaccines was determined. Percent was then calculated as (A∩B/sum(A,B)) * 100. Bars show mean values ± SEM. For the clusters shared between the two vaccine datasets, the frequency of the cluster, the average mutation of the cluster, and the diversity of the cluster was calculated for each dataset. (**b**–**d**) Changes in cluster frequency, mutation, and lineage diversity from the TIV vaccine dataset to the pH1N1 vaccine dataset. Boxes show locations of the 25, 50, and 75^th^ percentiles, and whiskers show data within 1.5x the interquartile range. For (**a**–**d**), p values represent the result from a two-sided t-test. (**e**) A representative lineage from both the non-adjuvanted (left) and AS03-adjuvanted (right) vaccine groups generated from the combined TIV and pH1N1 data. Each node in the lineage tree represents a unique sequence, and the size of the node represents the number of those sequences. The black node is the germline sequence, white nodes are inferred common ancestor sequences, and the colored nodes are those found in the datasets (purple = TIV only, orange = pH1N1 only and green = TIV and pH1N1). Numbers on the edges of adjoining nodes show the number of mutations separating the sequences.

Next, the effect of the adjuvant on recalled clusters was investigated. While mutated clusters are likely to represent those derived from memory recall, their exact antigenic specificity is unknown. To study recalled clusters that are likely to be influenza-specific, those present in the TIV dataset, that then re-appear in the pH1N1 dataset were found for each participant. As the probability of the same CDR3 being produced during two independent recombination events during the lifetime of a single individual is practically zero, it is likely that these shared clusters represent memory recall of the same B cell lineage^16^,^28^. On average, there were 52 clusters for each participant found in both the TIV and pH1N1 datasets, equating to approximately 1% of the total number of clusters present in the two datasets. This number was similar regardless of whether the adjuvanted or non-adjuvanted pH1N1 vaccine was given (Fig. 6a). The change in the properties of these clusters from the TIV to the pH1N1 dataset was then determined. Following the pH1N1 vaccination, there was an increase in average cluster size and mutation than from following TIV vaccination, but there was no difference between the adjuvanted and non-adjuvanted vaccine groups (Fig. 6b,c). Lineage trees were also generated from the clusters shared between the TIV and pH1N1 datasets. First, lineages were created just using the sequences from the TIV dataset, and then lineages were created using sequences from both the TIV and pH1N1 datasets. Adding in the pH1N1 sequences led to an increase in diversity of the lineages. This increase in diversity of the lineages was greater in the adjuvanted compared to the non-adjuvanted group (Fig. 6d, p  =  0.0211). Visualizing the lineages showed that in the non-adjuvanted group, the TIV and pH1N1 sequences were similar, which is why there was only a small increase in diversity (Fig. 6e; left side lineage). In the adjuvanted group, the pH1N1 sequences diverged more from the TIV sequences, which is what lead to the greater increase in diversity of these lineages (Fig. 6e; right side lineage).

### Adjuvant stimulates increased recall of cells with attributes of cross-reactivity

**Figure 7:**
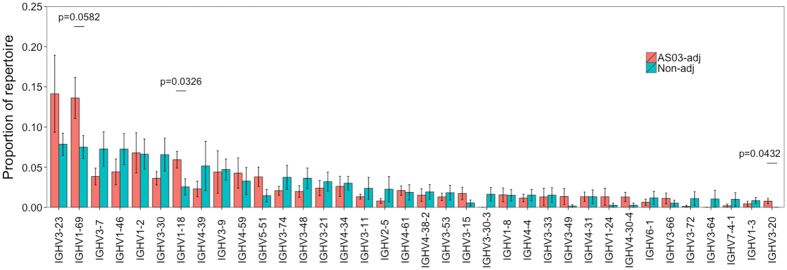
V gene usage of recalled clusters. For the clusters shared between the TIV and pH1N1 datasets, the proportion of these clusters utilizing different V gene segments was determined for each participant. Bars show mean values ± SEM. Shown are the V genes with a proportion above 0.005 in at least one of the vaccine groups. P values are shown for the three greatest differences between the adjuvanted and non-adjuvanted groups. Comparisons were performed using a two-sided t-test.

To further investigate properties of the recalled clusters between the TIV and pH1N1 vaccines, their V gene usage was determined. While biases in V gene use were broadly similar between the two vaccine groups, there were some differences between them (Fig. 7 and Table S2). The greatest group differences were in IGHV1-18 (p  =  0.0326), IGHV3-20 (p  =  0.0432), and IGHV1-69 (p  =  0.0582), which were used to a greater extent in the adjuvant compared to non-adjuvanted group. As IGHV1-69 is associated with HA stem binding cross-reactive antibodies, we next compared the recalled clusters to a database of 59 previously characterized cross-reactive HA stem binding monoclonal antibody sequences isolated from H1N1 vaccination studies^23^,^24^. Five of the recalled lineages contained sequences with the same CDR3 AA sequence, and V and J gene segment usage as those from the previous studies, and all of these lineages were present in participants receiving the adjuvanted vaccine only. From one of the published studies, the full VH nucleotide sequence was available^23^, so this could be compared to the sequences within the recalled lineages. Comparing the published sequence to its closest relative in each lineage indicated that there were only a small number of non-synonymous changes between them (12, 15 and 18 for the three lineages), so may not effect antigen-binding ability (Fig. S6). Critically, the CDR2 Phe residue at position 54, and the CDR3 Tyr residue at position 98, which are required for optimal binding^29^, remained unchanged.

### Adjuvant stimulates subclass switching to IgG1 and IgG3

**Figure 8:**
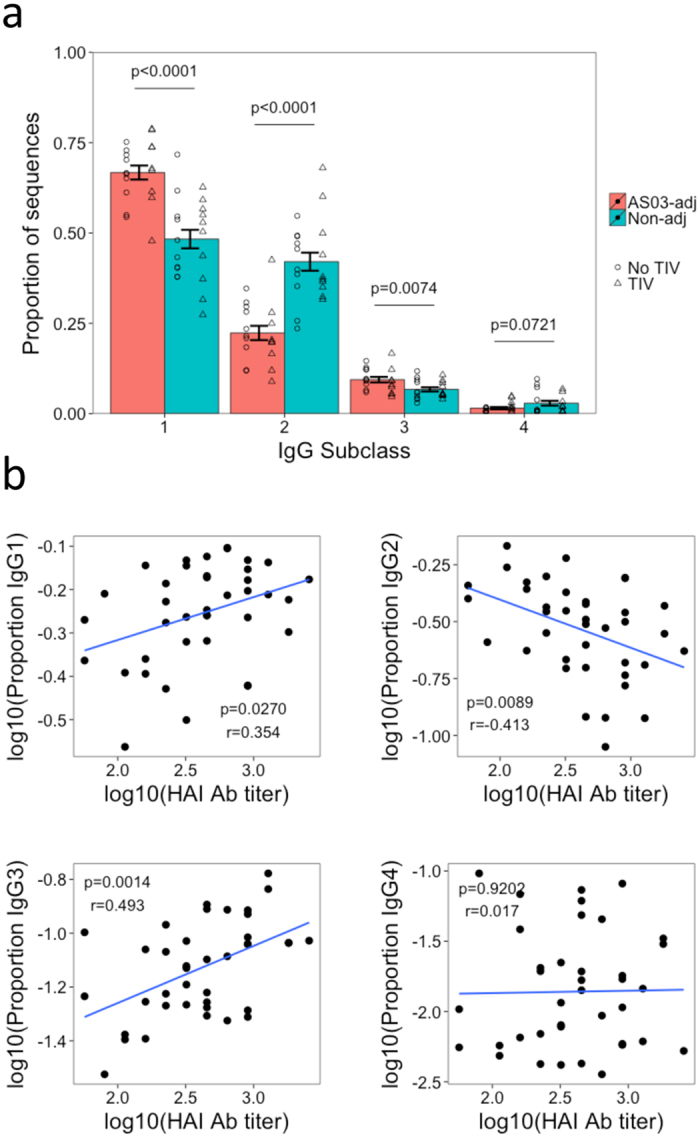
Analysis of IgG subclass usage. (**a**) For each participant, the proportion of sequences of each IgG subclass was determined. Bars show mean values ± SEM. P values represent the result from a two-sided t-test. Individual data points are shown, with the shape representative of whether the participant received the seasonal TIV prior to pH1N1 vaccination. (**b**) The proportion of the repertoire comprised by each of the four IgG subclasses was then correlated with the H1N1(California)-specific HAI antibody titer. R values show the Pearson product-moment correlation coefficient.

Subclass usage differed considerable between the vaccine groups (Fig. 8a). There was a higher proportion of IgG1 (P < 0.0001) and IgG3 (P = 0.0074) sequences, and a lower proportion of IgG2 (p < 0.0001) and IgG4 (p = 0.0721) sequences in the adjuvanted compared with the non-adjuvanted vaccine groups. These differences remained the same when considering only the mutated clusters, but the difference between the vaccine groups in IgG3 usage disappeared when considering only the unmutated clusters (Fig. S7).

The percent of sequences in the repertoire comprised of the different subclasses for each participant was then correlated with their vaccine response as measured by H1N1(California) HAI antibody titer (Fig. 8b). For both IgG1, and IgG3, the proportion of the repertoire comprised by these subclasses showed a moderate positive correlation with their vaccine response (p = 0.0270, r = 0.354 and p = 0.0014, r = 0.493 respectively). On the other hand, the proportion of the repertoire comprised by IgG2 sequences showed a negative correlation with vaccine response, while the proportion of the repertoire comprised by IgG4 sequences showed no correlation with vaccine response. The adjuvanted vaccine therefore promoted increased generation of IgG1 and IgG3, which correlated with an improved vaccine response.

## Discussion

In this study, high-throughput sequencing of the plasma cell BCR repertoire was applied for detailed investigation of the effect of the AS03 adjuvant on pH1N1 influenza vaccination. We found that the increased immunogenicity of the adjuvanted vaccine could potentially be explained by both an increase in the activation of naïve B cells, as well as an increase in the adaptation of pre-existing memory B cells. There were a number of differences in the repertoire caused by the adjuvant, which could be used to distinguish the response to the two vaccines, and back up these findings.

A previous study of the plasma cell response following seasonal influenza vaccine showed that the activated vaccine-specific plasma cells have high levels of mutation, indicating that they are likely derived from memory recall, and not activation of naïve B cells^17^. These plasma cells were still specific for the current vaccine antigen, indicating that the response to influenza tends to be dominated by memory recall, and that the recalled cells can then fine-tune their specificity through further mutation^17^. In the context of pandemic vaccine, where the difference between the vaccine strain and previously encountered strains is greater, this fine-tuning of specificity of the recalled cells may not be as efficient, thus leading to immune interference through original antigenic sin, and a reduction of vaccine immunogenicity^11^. Indeed, previous studies have shown that prior receipt of TIV does reduce the subsequent response to pH1N1 vaccination^31^,^32^. We observed in this study that following adjuvanted vaccine there is an increase in the number of plasma cells with low mutation levels, indicative of recent activation from naïve B cells. This suggests that the adjuvant potentially increases immunogenicity by overcoming this immune interference by instead activating more naïve B cells specific to the new vaccine antigen. While we cannot be certain of the antigenic-specific of all the plasma cells that we isolated in this study, we show here that they are enriched for vaccine-specificity (Fig. 3), and previous studies have shown that 7 days following administration of pH1N1 vaccine, 33–80% of plasma cells isolated are specific to the vaccine^17^,^18^. Furthermore, searching for previously described pH1N1-specific BCR sequences in our dataset shows that they can be found in both the mutated and unmutated clusters. Annotating specificity of sequences in our dataset based on previously described antigen-specific sequences gives great analytical potential, but it should be noted that these assumptions of specificity couldn’t be functionally confirmed. As techniques for high-throughput sequencing of paired BCR heavy and light chains improve, future studies will be able to assess binding specificity directly^33^.

The distinct V gene usage profile seen when comparing the unmutated and mutated plasma cells may relate to distinct epitopes targeted by these two populations. There have been numerous studies showing that certain V genes are preferentially used in the response to different antigens or epitopes^14^. In the context of influenza, IGHV1-69 has been well characterized as being important in the production of HA stem-binding cross-reactive antibodies^29^. The reduction in IGHV1-69 among other V gene segments in the unmutated plasma cell sequences may therefore relate to a decreased tendency of these sequences to bind the HA stem, although we are unable to functionally confirm this hypothesis using data from the current study, and IGHV1-69 is also used in the response to other antigens^34^. It is interesting to note though that Khurana *et al*. have also made a similar observation, showing that the MF59 adjuvant stimulated a switch away from stem binding towards head binding specificity^12^,^13^.

While the adjuvant did appear to increase the proportion of unmutated plasma cells likely derived from naïve B cells, these still formed a minority of the total plasma cell response, so it is likely that the adjuvant functions through other mechanisms as well. When the recalled clusters between the TIV and pH1N1 vaccines were specifically studied, it was found the adjuvant stimulated greater diversification of the lineages. This is consistent with the adjuvant having a greater ability to fine-tune the specificity of the lineages through further rounds of affinity maturation^17^. This increased ability of the adjuvant to drive re-diversification may be what enables the adjuvant to overcome the interference effects from prior receipt of TIV^31^,^32^. It was further found that some of the recalled clusters in the adjuvant group had properties consistent with them being derived from cross-reactive B cells that bound the HA stem. While the pH1N1 vaccine has previously been seen to induce cross-reactive stem binding B cells^24^, we show here that this ability appears to be enhanced if the vaccine is adjuvanted.

It should be noted that there are some limitations to our methodology that should be taken into account when interpreting the lineage data. PCR introduces amplification bias as well as artifacts due to error and chimera formation. These artifacts may lead to an artificial inflation of lineage diversity, but will equally impact all lineages, so should not affect the comparisons drawn here. In the future, the impact of such artifacts could be reduced by the incorporation of unique molecular identifiers during the reverse transcription step^16^.

The AID enzyme, which drives somatic hypermutation and affinity maturation, and may therefore be responsible for lineage diversification, also drives isotype class switching^27^. Specifically, switching to both IgG1 and IgG3 is related to TNF-α and IL-6 production, and this also correlates with the production of AID^35^. It has previously been shown that IgG3 is the most important subclass used in the H1N1 response^35^,^36^. Focusing on IgG3 in our dataset showed this to be more prevalent following adjuvanted vaccine, and that there was a positive correlation between the proportion of IgG3 sequences in the repertoire, and the HAI Ab titer. It may be that the Fc receptor of IgG3 is more effective at neutralizing influenza than other subclasses^37^, or that the subclass switching is just a proxy for AID production, and it is the AID mediated lineage diversification which improves the neutralizing ability of the antibodies.

The data presented here support the use of the adjuvant for increasing the immunogenicity of influenza vaccines, particularly where the vaccine is highly different to previously encountered strains and thus requires increased naïve B cell activation, and/or increased adaptation of pre-existing memory B cells. Furthermore, this study demonstrates the utility of BCR sequencing for understanding vaccine responses, and the benefit of having previously described antigen-specific sequence data with which to annotate these datasets.

## Materials and Methods

### Study design

Of the 118 participants included in the original study^8^, samples from a random subset of 39 participants were used for this study. The study (clinicaltrials.gov; NCT01059617) was conducted at three study centers in the United States, after approval of the protocol by an independent local ethics committee (Chesapeake Research Review; 0004657). The study was undertaken in accordance with the Helsinki Declaration and good clinical practices. All participants provided informed written consent before entering the study. The participants were healthy, aged between 19–40 years, and had no prior immunisation history with the 2009 pH1N1 vaccine. Participants were randomized 1:1:1:1 to four study groups: A, B, C and D (Fig. 1). For this study, we had 10 participants from groups A, C and D, and 9 participants from group B. Group A and B were administered TIV, while groups C and D were administered saline at day 0. Samples were taken for plasma cell sorting 7 days following TIV vaccination in groups A and B. Four months later, two doses of pH1N1 vaccine were given to all participants at a three-week interval. Groups A and C received AS03-adjuvanted vaccine, while groups B and D received non-adjuvanted vaccine. Samples were taken for plasma cell sorting and immunogenicity evaluations 7 days following the second pH1N1 vaccine.

### Vaccines

GlaxoSmithKline, Quebec, Canada, manufactured vaccines. The TIV was the 2009–2010 vaccine, containing 15 μg HA each of A/Brisbane/59/2007(H1N1) IVR-148, A/Uruguay/716/2007(H3N2) NYMCX-175C and B/Brisbane/60/2008(B). The pH1N1 vaccine was the 2009 pandemic vaccine containing HA of A/California/7/2009(H1N1). The non-adjuvanted vaccine contained 15 μg HA. The adjuvanted vaccine contained 3.75 μg HA with the AS03_A_ oil-in-water emulsion adjuvant.

### Immunogenicity evaluations

Methods for the immunogenicity evaluations carried out in this study are detailed in the previous publication^8^. Immunogenicity evaluations were carried our 7 days following administration of the second pH1N1 vaccine. Briefly, HAI titers were measured as the highest serial dilution of serum that prevented hemagglutination. Seropositivity was defined as a titer ≥1:10.

Memory B cell ELISpot was used to determine frequencies of H1N1(California)-specific memory B cells. Cells were induced to differentiate into plasma cells by incubating in culture medium containing CpG DNA for 5 days prior to addition to an antigen-coated ELISpot plate. Memory B cell ELISpot was only conducted for a subset of the participants in the original study, which equated to 17 of the 39 participants included in this study.

Activated H1N1(California)-specific CD4 T cell numbers were assessed using intracellular cytokine staining, followed by flow cytometry. To be classed as activated, the T cells were required to express at least two of the following immune markers: interferon-gamma, interleukin-2, tumor necrosis factor-alpha and CD40 ligand^8^.

### Plasma cell sorting

PBMC’s were first isolated from whole blood by density-gradient centrifugation, and stored at −80 °C prior to use. Samples were defrosted at 37 °C, and washed twice with RPMI supplemented with 10% fetal calf serum, penicillin, streptomycin, L-glutamine, and benzonase. Cells were then stained with CD3-pacific blue, CD19-FiTC, CD20-PECya7, CD27-APC and CD38-PerCPCya5.5. CD3−, CD19+, CD20−, CD27+ and CD38+ plasma cells (Fig. S1) were sorted using a FACSAria cell sorter, and resuspended in RLT lysis buffer (Qiagen). Samples were snap-frozen, and stored at −80 °C.

### BCR sequencing

Following defrosting, RNA was extracted from sorted cells using the RNeasy Mini Kit (Qiagen). cDNA was generated using random hexamer primers, and the SuperScript III (Invitrogen) enzyme kit (42 °C for 60 min, 95 °C for 10 min). IgG transcripts were amplified using the Multiplex PCR kit (Qiagen), using 200 nM each of 6 V family specific forward primers, and an IgG reverse primer (94 °C for 15 min, 33 cycles of 94 °C for 30 s, 58 °C for 90 s and 72 °C for 30 s, and 72 °C for 10 min)^38^. Amplicons were gel extracted and purified, and then multiplexed for sequencing on a single 2 × 300 bp MiSeq (Illumina) run.

### Sequence Processing

Paired-end reads were joined using fastq-join (ea-utils) using default setting, and filtered for a minimum Phred score of 30 over 75% of bases. IMGT/HighV-Quest^39^ was used for VDJ assignment and sequence annotation. Sequences defined as unproductive by IMGT were removed. Sequences were independently clustered for each sample to group together those arising from PCR error, and also to group together those arising from clonally related B cells. The clustering required identical V and J segment use, identical CDR3 length, and allowed 1 AA mismatch for every 12 AA’s in the CDR3; this threshold was validated as part of a previous study^15^,^19^. For determining clusters shared between the TIV and pH1N1 datasets, samples from the same participant were clustered together, and a cluster defined as shared if at least 1 sequence was contributed from each dataset.

To filter for clusters comprised by sequences from multiple B cells, and not just sequences arising from PCR error, only clusters containing at least 50 sequences were used to create lineage trees. Subsampling down to 50 sequences was performed if there were more. Lineage trees were created using the alakazam software package^40^.

### Statistical analysis and graphing

Statistics were performed using R^41^; specific tests used are detailed in the figure legends. Repertoire diversity, and lineage diversity were calculated using the Shannon entropy index, treating either individual clusters, or individual sequences within the clusters as species respectively. Principal component analysis was performed using the prcomp function in R. Graphs were created using ggplot2, and lineage trees visualized using the iGraph R packages.

## Additional Information

**How to cite this article**: Galson, J. D. *et al*. Investigating the effect of AS03 adjuvant on the plasma cell repertoire following pH1N1 influenza vaccination. *Sci. Rep.* **6**, 37229; doi: 10.1038/srep37229 (2016).

**Accession Codes:**: The BCR sequence dataset supporting the conclusions of this article can be obtained from the NCBI Sequence Read Archive under the accession number SRP080786.

**Publisher’s note:** Springer Nature remains neutral with regard to jurisdictional claims in published maps and institutional affiliations.

## Supplementary Material

Supplementary Information
