# Supplementary material for: Investigating the effect of AS03 adjuvant on the plasma cell repertoire following pH1N1 influenza vaccination
Source: Sci Rep. 2016 Nov 16;6:37229. doi: 10.1038/srep37229 (PMC5110968; doi:10.1038/srep37229)
Supplement: Supplementary Information [file srep37229-s1.pdf]

Supplementary Information

Investigating the effect of AS03 adjuvant on the plasma cell repertoire following pH1N1 influenza vaccination

J. D. Galson, J. Trück, D. F. Kelly, & R. van der Most

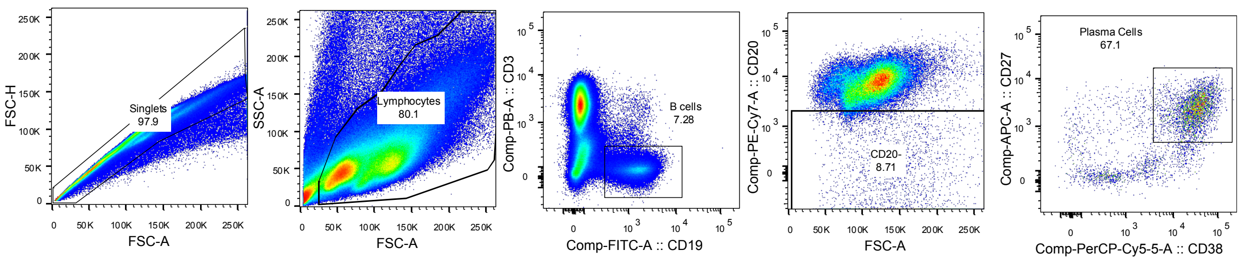

Fig. S1. Cell sorting gating strategy. Doublets were gated out, and lymphocytes gated based on forward scatter and side scatter. B cells were identified as the CD3-, CD19+ lymphocyte population, and plasma cells were then identified as the CD20-, CD27+ and CD38+ B cell population.

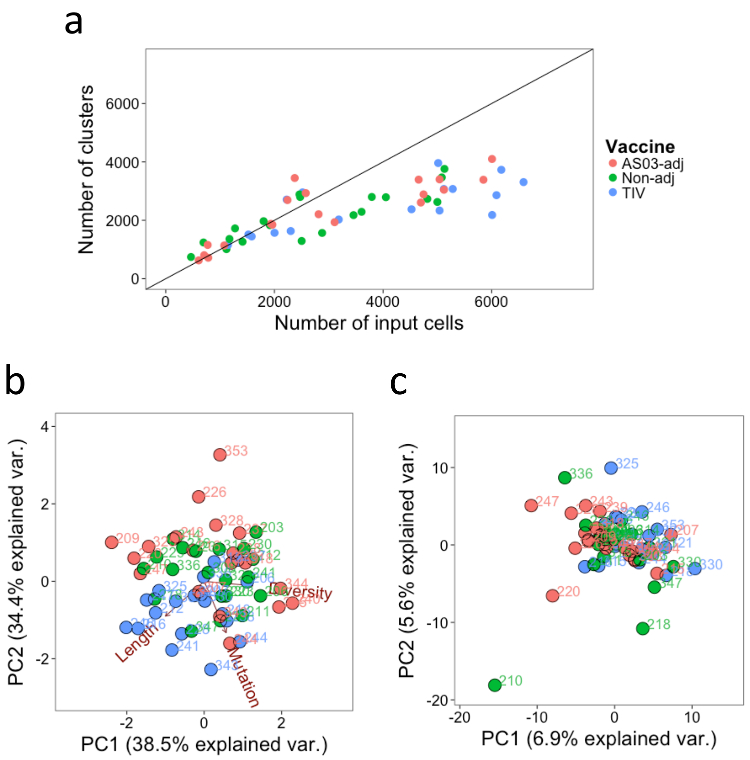

Fig. S2. BCR repertoire quality control measures for plasma samples after each vaccine. (**a**) Relationship between the number of plasma cells isolated from each sample, and the number of clusters generated from the sequence data of that sample. Line represents x=y. (**b**) Principle component analysis based on general repertoire properties of Shannon diversity, mutation, and CDR3 sequence length. (**c**) Principle component analysis based on the proportion of the repertoire comprised by clusters utilizing different V gene segments.

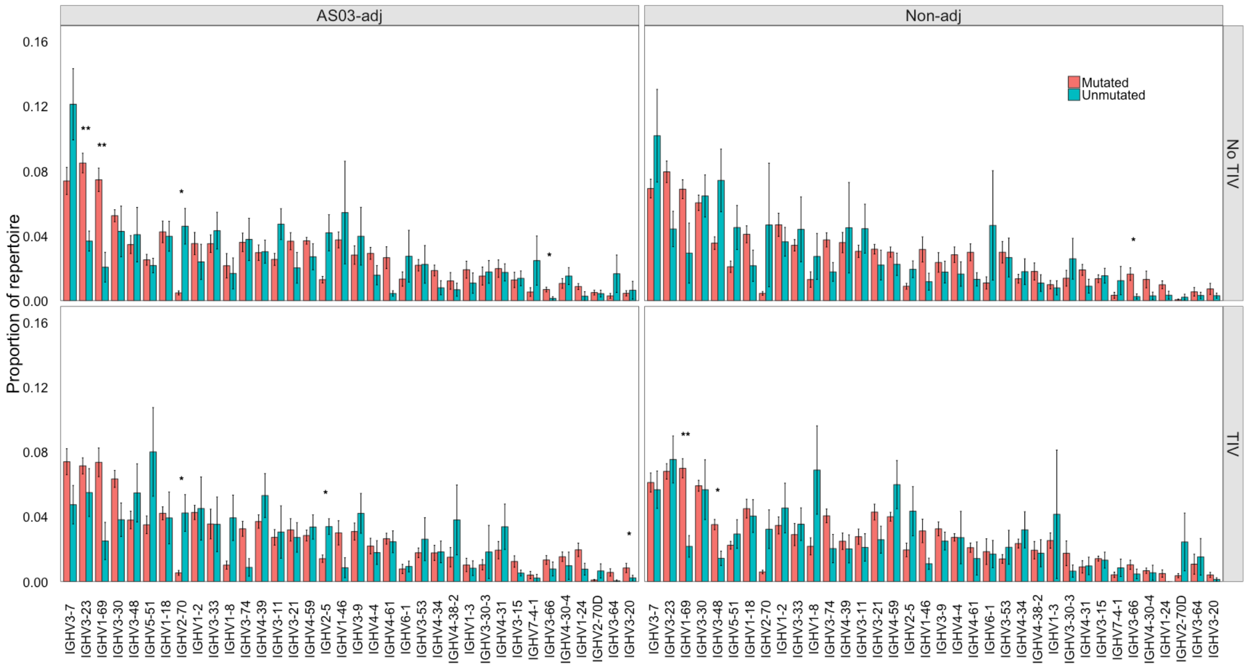

Fig. S3. Comparing V gene usage of unmutated naïve clusters to mutated clusters derived from memory recall split by vaccine group. For each sample, the proportion of both mutated and unmutated clusters utilizing different V gene segments was determined, and this was split by whether adjuvanted or non-adjuvanted vaccine was given, and whether there was prior TIV vaccination. Bars show mean values ± SEM. Shown are the V genes with a proportion above 0.005 in at least one of the groups. Comparisons were performed using a two-sided paired t-test. * $p < 0.01$, ** $p < 0.001$, *** $p < 0.0001$

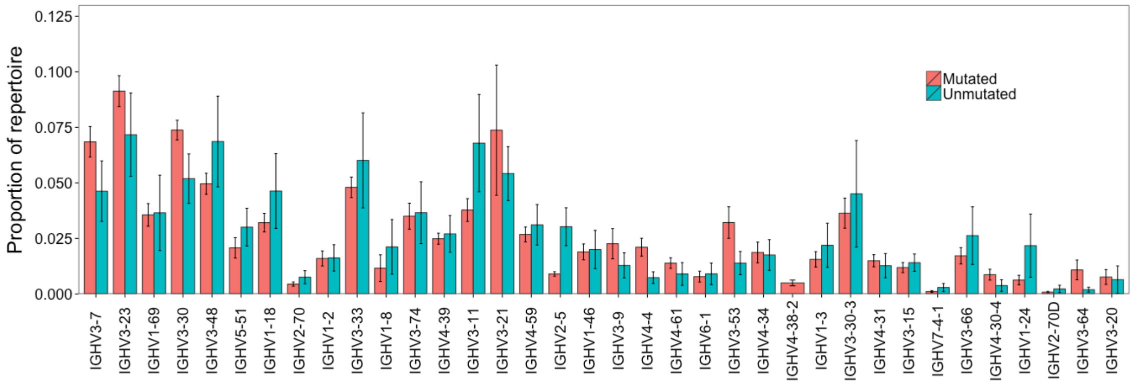

Fig. S4. Comparing unmutated and mutated clusters derived from plasma cells in the absence of a specific immune stimulus. For each sample, the proportion of both mutated and unmutated clusters utilizing different V gene segments was determined. V gene ordering on the y axis is the same as shown in Fig. 4. N=14.

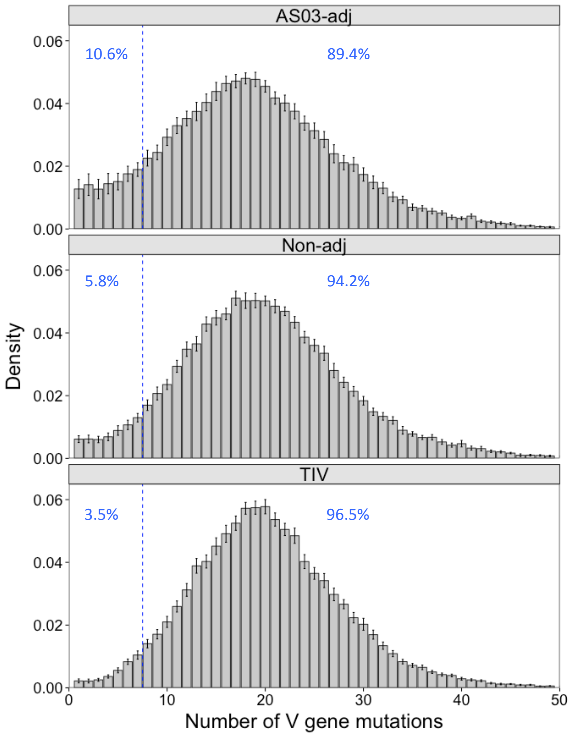

Fig. S5. Distribution of clusters with different mean numbers of V gene mutations, split by vaccine group. Dotted vertical line separates clusters defined as unmutated or mutated (threshold of 7). Bars show mean values ± SEM.

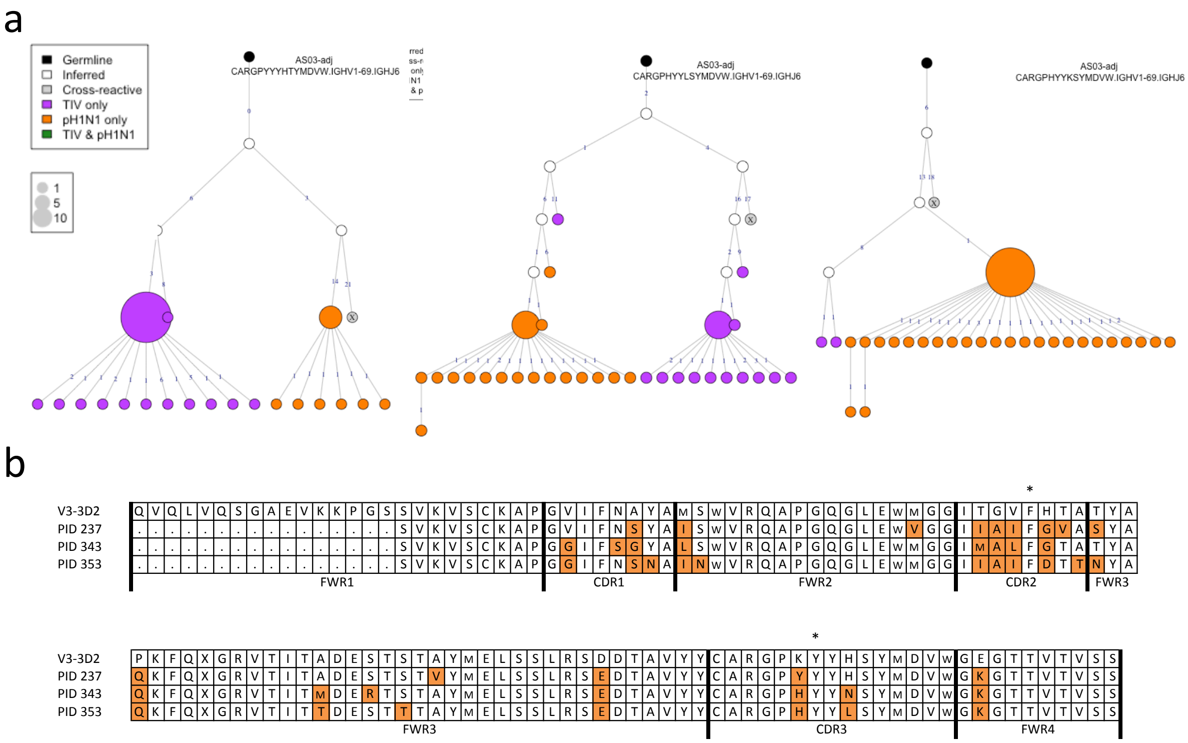

Fig. S6. Similarity of the previously described cross-reactive sequence (V3-3DT) to sequences in our dataset. (**a**) Lineages generated from the combined TIV and pH1N1 data with similarity to V3-3DT. The V3-3DT sequence is included in the lineage generation. Each node in the lineage tree represents a unique sequence, and the size of the node represents the number of those sequences. The black node is the germline sequence, white nodes are inferred common ancestor sequences, the colored nodes are those found in the datasets (purple = TIV only, orange = pH1N1), and the grey node with the "X" is the V3-3DT sequence. Numbers on the edges of adjoining nodes show the number of mutations separating the sequences. (**b**) Alignment of the V3-3DT AA sequence to the closest match from the three lineages. Orange squares represent differences. * Residues with particular importance for antigen binding.

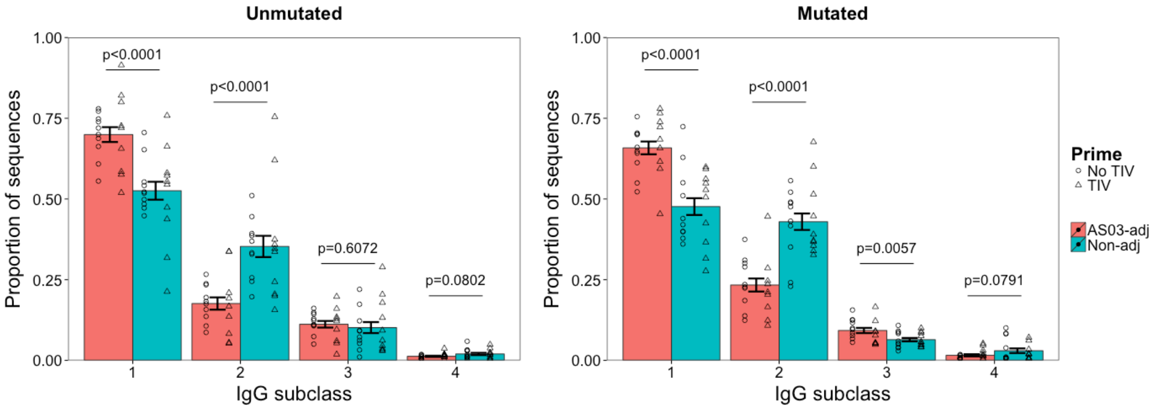

Fig. S7. Analysis of IgG subclass usage split by mutated and unmutated sequences. For each participant, the clusters were split according to mutation level (less than 8 mutations = unmutated, and more than 8 = mutated), and the proportion of sequences of each IgG subclass was determined. Bars show mean values ± SEM. P values represent the result from a two-sided t-test. Individual data points are shown, with the shape representative of whether the participant received the seasonal TIV prior to pH1N1 vaccination.

Table S1. Number of cells, raw sequence reads, filtered sequence reads, and clusters obtained for each plasma cell sample.

Participant	Vaccine	Cell number	Raw Sequences	Filtered sequences	Clusters
207	AS03-adj	1963	311089	144242	1849
237	AS03-adj	4745	381734	193031	2888
238	AS03-adj	2241	329443	164355	2693
244	AS03-adj	1933	315791	155593	1879
247	AS03-adj	606	343785	178377	627
343	AS03-adj	3108	310611	150935	1935
353	AS03-adj	5121	300489	161379	3046
216	AS03-adj	2813	286853	139327	2207
325	AS03-adj	707	355057	189135	804
209	AS03-adj	782	318189	160831	718
215	AS03-adj	6006	383915	190962	4099
220	AS03-adj	771	326679	161972	1156
226	AS03-adj	5848	336676	170909	3388
239	AS03-adj	4696	227580	112361	2608
240	AS03-adj	4655	325885	153925	3392
328	AS03-adj	2577	290798	136686	2931
344	AS03-adj	5040	287659	149208	3402
243	AS03-adj	1076	374145	150293	1138
318	AS03-adj	2374	365403	184945	3448
206	Non-adj	4815	358695	173032	2733
212	Non-adj	3605	347768	167570	2290
218	Non-adj	2501	338607	151708	1292
228	Non-adj	1275	302111	138733	1722
241	Non-adj	3793	320134	162093	2799
246	Non-adj	4052	340498	168373	2790
315	Non-adj	1916	339940	176107	1832
321	Non-adj	3456	330906	162663	2177
330	Non-adj	5000	301945	144793	2627
336	Non-adj	1117	253429	142586	1012
203	Non-adj	2474	268607	126200	2797
210	Non-adj	464	371013	193104	743
211	Non-adj	694	266428	129160	1241
223	Non-adj	1172	342051	156535	1361
229	Non-adj	1413	335175	160619	1266
230	Non-adj	2461	264889	136836	2885
231	Non-adj	1801	320018	162852	1970
308	Non-adj	5130	369861	178008	3760

314	Non-adj	2878	382207	204088	1567
347	Non-adj	5082	378231	185098	3469
206	TIV	6177	386565	190146	3729
212	TIV	3184	282988	143753	2027
218	TIV	4524	331594	174269	2377
228	TIV	1521	265217	135653	1513
241	TIV	5040	334283	169037	2334
246	TIV	6008	284785	148185	2182
315	TIV	2002	320567	144717	1571
321	TIV	5119	358420	174237	3072
330	TIV	2518	346997	184586	2956
336	TIV	10651	377916	185996	3522
207	TIV	6088	341042	184384	2860
237	TIV	5015	395822	197720	3960
238	TIV	6586	402635	196310	3307
244	TIV	2225	290349	135800	2710
247	TIV	2300	359212	178606	1632
343	TIV	10003	382209	196999	4528
353	TIV	5283	399934	212514	3073
216	TIV	1582	273374	142088	1447
325	TIV	1144	263354	141696	1155

Table S2. Differences in V gene usage of the recalled sequences between the adjuvanted and non-adjuvanted vaccine groups. P value represents the result from a two-sided t test.

V gene	P value
IGHV1-18	0.032640406
IGHV3-20	0.043151999
IGHV1-69	0.058279113
IGHV3-30-3	0.08134377
IGHV4-30-4	0.128422804
IGHV5-51	0.130176845
IGHV3-7	0.175057635
IGHV3-73	0.180154569
IGHV3-15	0.189379996
IGHV3-43	0.190123149
IGHV4-28	0.190123149
IGHV3-30	0.208455681
IGHV3-23	0.238371259
IGHV3-49	0.268385833
IGHV1-46	0.275295246
IGHV3-48	0.283181036
IGHV3-72	0.286007224
IGHV3-74	0.319574
IGHV3-13	0.343436396
IGHV3-43D	0.343436396
IGHV3-64	0.343436396
IGHV5-10-1	0.343436396
IGHV1-69-2	0.346593507
IGHV2-70	0.346593507
IGHV3-64D	0.346593507
IGHV1-24	0.354672875
IGHV7-4-1	0.376604538
IGHV2-5	0.383093744
IGHV4-39	0.392086809
IGHV1-3	0.436852103
IGHV3-66	0.452579118
IGHV3-11	0.481378339
IGHV6-1	0.559274072
IGHV3-21	0.600622076
IGHV1-58	0.620474415
IGHV3-53	0.622104274
IGHV4-4	0.668144457
IGHV3-NL1	0.696850906

IGHV4-59	0.700206038
IGHV4-38-2	0.74353451
IGHV4-34	0.799007524
IGHV4-61	0.848269843
IGHV4-30-2	0.858577832
IGHV3-33	0.877714457
IGHV3-9	0.917894644
IGHV1-2	0.958548807
IGHV1-8	0.966585141
IGHV4-31	0.995102861
